# Bacterial diversity and functional analysis of severe early childhood caries and recurrence in India

**DOI:** 10.1038/s41598-020-78057-z

**Published:** 2020-12-04

**Authors:** Balakrishnan Kalpana, Puniethaa Prabhu, Ashaq Hussain Bhat, Arunsaikiran Senthilkumar, Raj Pranap Arun, Sharath Asokan, Sachin S. Gunthe, Rama S. Verma

**Affiliations:** 1 Department of Biotechnology, Indian Institute of Technology Madras, Chennai, Tamil Nadu India; 2 Department of Civil Engineering, Indian Institute of Technology Madras, Chennai, Tamil Nadu India; 3 Department of Biotechnology, K. S. Rangasamy College of Technology, Tiruchengode, Tamil Nadu India; 4 Department of Pediatric Dentistry, K. S. R Institute of Dental Science and Research, Tiruchengode, Tamil Nadu India; 5 Department of Biotechnology, Bhupat and Jyoti Mehta School of Biosciences, Indian Institute of Technology Madras, Block 1, Room No. 201, Chennai, 600036 India

**Keywords:** Next-generation sequencing, Bioinformatics, Biofilms, Microbial communities, Infectious-disease diagnostics, Biochemical reaction networks, Classification and taxonomy

## Abstract

Dental caries is the most prevalent oral disease affecting nearly 70% of children in India and elsewhere. Micro-ecological niche based acidification due to dysbiosis in oral microbiome are crucial for caries onset and progression. Here we report the tooth bacteriome diversity compared in Indian children with caries free (CF), severe early childhood caries (SC) and recurrent caries (RC). High quality V3–V4 amplicon sequencing revealed that SC exhibited high bacterial diversity with unique combination and interrelationship. *Gracillibacteria_GN02* and TM7 were unique in CF and SC respectively, while *Bacteroidetes, Fusobacteria* were significantly high in RC. Interestingly, we found *Streptococcus oralis* subsp. *tigurinus* clade 071 in all groups with significant abundance in SC and RC. Positive correlation between low and high abundant bacteria as well as with TCS, PTS and ABC transporters were seen from co-occurrence network analysis. This could lead to persistence of SC niche resulting in RC. Comparative in vitro assessment of biofilm formation showed that the standard culture of *S. oralis* and its phylogenetically similar clinical isolates showed profound biofilm formation and augmented the growth and enhanced biofilm formation in *S. mutans* in both dual and multispecies cultures.

## Introduction

Interaction among more than 700 species of microbiota under different micro-ecological niches of the human oral cavity^1,2^ acts as a primary defense against various pathogens. This has been observed to play a significant role in child’s oral and general health. Dysbiosis among these microbes due to excessive and frequent intake of carbohydrates results in acidic niche, thereby lowering the buffering provided by healthy microbiome. This condition leads to demineralization of tooth surface causing caries in children^3,4^. Once lesions advance beyond the white spot stage and the enamel surface is damaged, they cannot be biologically reversed resulting in Severe Early Childhood Caries (SC) among the children with the age group of 3–6 years^5^. Considerable change in persistent oral biota in response to the functional molecules even after the treatment of SC facilitates recurrent caries (RC) in children, affecting both primary and permanent dentition^6^.

Moreover, dysbiosis of oral microbes also results in acute to chronic disease conditions either directly or indirectly by producing metabolically active compounds that interrupt host immune system. Various studies have shown that harmful oral microbiome may hold a significant impact beyond the oral cavity that is related to systemic diseases^7^, including elevated cardiovascular risk^8,9^, rheumatoid arthritis^10^, adverse pregnancy outcome^11^ and digestive diseases^12^. Such factors make it imperative to know the colonization patterns of oral commensals occurring during childhood and their benign impact in oral and systemic diseases and health conditions.

Metagenomics studies with the help of high-throughput sequencing technologies revolutionized the human microbiome research^13^, providing an opportunity to tap and focus on the unexplored complex microbial systems that are difficult to cultivate in-vitro. Increased understanding about the functional activity of microbes within the complex microbial community with that of host ecosystems was possible with the advancement of computational analysis tools for these sequences^14^. According to Keystone Pathogen Hypothesis^15^, understanding and identifying the complex interactions between high and low abundant microbes and its functional potential that resist any therapeutic agents under the micro-ecological niche. Such analysis may prevent an adverse effect on the human system and can become a new target for treatment and preventive care for caries as well as other related diseases.

Here, we report the analysis of both over-represented and under-represented caries causing microbiota, its predictive functional traits, co-aggregation with each other and their susceptibility towards secondary caries in SC and RC of Indian children. Importance was given to the exploration of biomarkers in-order to identify the key pathogen and its functional properties that resist the micro ecological stress. Interaction network analysis among the oral microbiota and functional traits would enhance the understanding about the strong network of oral bacterial species especially in SC and RC that could paves way for early detection and management of dental caries.

## Results

### Demography of caries status

In the current report, study subjects were categorized into three study groups and two gender groups. Caries status of the participants, their age and gender are presented in Table 1. Sequencing were performed for 30 samples, Caries Free (CF) (n = 10), Severe Early Childhood Caries (SC) (n = 10), Recurrent Early Childhood Caries (RC) (n = 10), from the 55 children enrolled because of low yield and quality of DNA. Gender proportion (Female/Male) of the study subjects CF, SC and RC were 5/5, 4/6, 6/4 respectively. Significant differences were not observed in the mean age and gender proportion among three study groups.

**Table 1:** Patient demographic data and QC of NGS data.

Sample ID	Study group	Gender	Age	DMFT	No. of sequences	Unique reads	Duplicate reads	% Trimmed	% of top overrepresented sequence	% of the sum of remaining overrepresented sequences
P1	Normal	Female	3	0	324,653	30,867	293,786	0.029	13.8	58.08
P2	Normal	Female	6	0	368,052	34,579	333,473	0.03	8.2	64.01
P3	Normal	Female	5	0	500,131	51,239	448,892	0.025	11.7	56.07
P8	Normal	Female	3	0	843,584	154,048	689,536	0.032	17.4	51.24
P9	Normal	Female	4	0	426,340	39,929	386,411	0.029	12.4	57.52
P10	Normal	Male	5	0	791,013	69,365	721,648	0.035	1.6	65.50
P11	Normal	Male	4	0	1,044,615	87,922	956,693	0.033	2.1	57.34
P15	Normal	Male	4	0	989,960	91,168	898,792	0.035	2.7	61.17
P17	Normal	Male	5	0	959,351	83,279	876,072	0.034	2.1	63.31
P4	Disease	Male	5	5	581,388	62,484	518,904	0.031	8.3	58.69
P5	Disease	Female	4	4	541,995	46,244	495,751	0.033	7.1	64.15
P6	Disease	Female	5	4	763,526	53,837	709,689	0.033	10.8	65.19
P7	Disease	Male	4	4	519,897	44,592	475,305	0.03	17.0	59.49
P13	Disease	Female	5	4	928,077	67,115	860,962	0.036	4.4	68.04
P14	Disease	Male	6	5	790,177	38,068	752,109	0.038	16.4	64.44
P33	Disease	Male	5	5	423,672	37,885	385,787	0.031	13.0	58.97
P35	Disease	Male	6	5	976,071	65,141	910,930	0.034	2.1	61.96
P39	Disease	Male	6	5	289,569	30,562	259,007	0.031	6.2	65.03
P40	Disease	Female	5	4	400,603	43,049	357,554	0.03	6.9	61.39
P19	Recurrent	Male	6	2	1,197,632	76,828	1,120,804	0.037	19.5	60.19
P21	Recurrent	Female	7	2	963,211	69,289	893,922	0.035	3.8	59.44
P23	Recurrent	Male	6	2	946,556	91,744	854,812	0.033	2.4	58.57
P25	Recurrent	Female	7	3	916,099	85,611	830,488	0.033	3.8	57.66
P31	Recurrent	Female	7	3	980,648	69,794	910,854	0.036	3.7	66.27
P32	Recurrent	Female	6	4	1,018,812	56,302	962,510	0.037	9.6	73.01
P36	Recurrent	Female	7	3	423,917	39,608	384,309	0.03	8.0	63.27
P37	Recurrent	Female	7	2	332,321	44,845	287,476	0.031	9.1	58.15
P38	Recurrent	Male	6	3	343,591	38,805	304,786	0.029	7.3	61.88

### Biodiversity of microbiota among SC, RC and CF micro ecosystem through high quality sequencing

**Figure 1:**
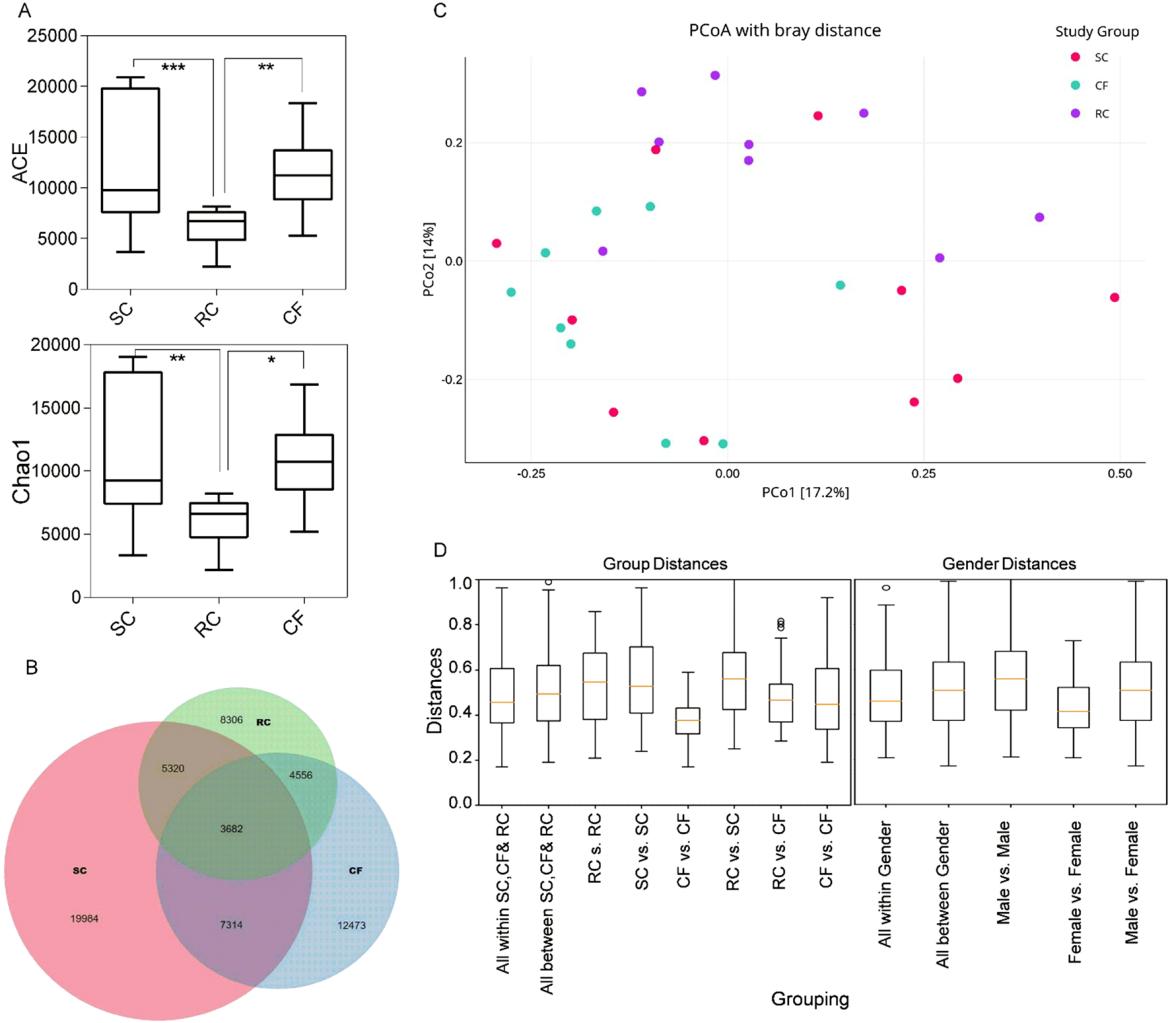
Diversity comparison of SC, CF and RC microbiota. (**A**) Boxplot of ACE and Chao 1 diversity in the three groups. Outliers are represented by dots. Significant differences between groups are shown by lines and the following notation. P < 0.05 (*), P < 0.01 (**), P < 0.001 (***). (**B**)Venn diagram illustrated the number of shared OTUs between SC, CF and RC at 97% similarity. Coloured circles represented each group and intersection part between circles represented the number of shared OTUs. (**C**) Principal coordinates analysis (PCoA) based on weighted UniFrac distance of community structure among all the individuals of three treatment groups. Each color circle represents one sample: pink for SC group (n = 10); blue for CF group (n = 8); violet for RC group (n = 10). (**D**) Box plot comparison of beta diversity analysis across treatment groups at genera level. The X-axis denotes the population studied while Y-axis denotes the corresponding Shannon–Weaver index representing the beta diversity of treatment and gender group.

A total of 15,270,361 high quality reads were generated from 28 samples with an average of 391,573 per sample ranging between 110,367 and 772,880 after trimming out low quality reads (Table 1). High richness in species diversity using Shannon, Chao and ACE index with 52,628 unique OTU’s after trimming out the low-quality reads were observed. RC exhibits significant difference in Chao 1 index with CF (P = 0.039), SC (P = 0.043) and in ACE with CF (0.012) and SC (P = 0.009) (Fig. 1A). Out of 52,628 OTU’s obtained in the analysis SC, CF and RC are characterized by 19,984, 12,473 and 8306 unique OTU’s respectively. SC and CF shared 7314 OTU, CF and RC shared 4556, RC and SC shared 5320 OTU’s. In common 3682 OTU’s were shared by all three study groups. Representative Venn diagram illustrates the number of unique and shared OTUs in each study group (Fig. 1B). To investigate the relatedness of microbiome composition, separation of beta diversity among CF, SC and RC samples based on PCoA (Principle Co-ordinates) axes 1 and 2 was given in the Bray Curtis Plot (Fig. 1C). The microbiota shows clear segregation between CF and RC but between CF–SC and SC–RC the microbiota overlaps considerably. Boxplot analysis of Weighted UniFrac distance showed significant difference between all groups and within group comparison except the distance between SC vs SC to RC vs RC and SC vs SC to all within the study group and between study groups (Fig. 1D). Similarly, significant differences were observed for all with in gender to male vs male and female vs female (Fig. 1D).

### Taxonomical analysis of bacterial diversity and its phylogenetic relationship in SC, RC and CF group of Indian children

**Figure 2:**
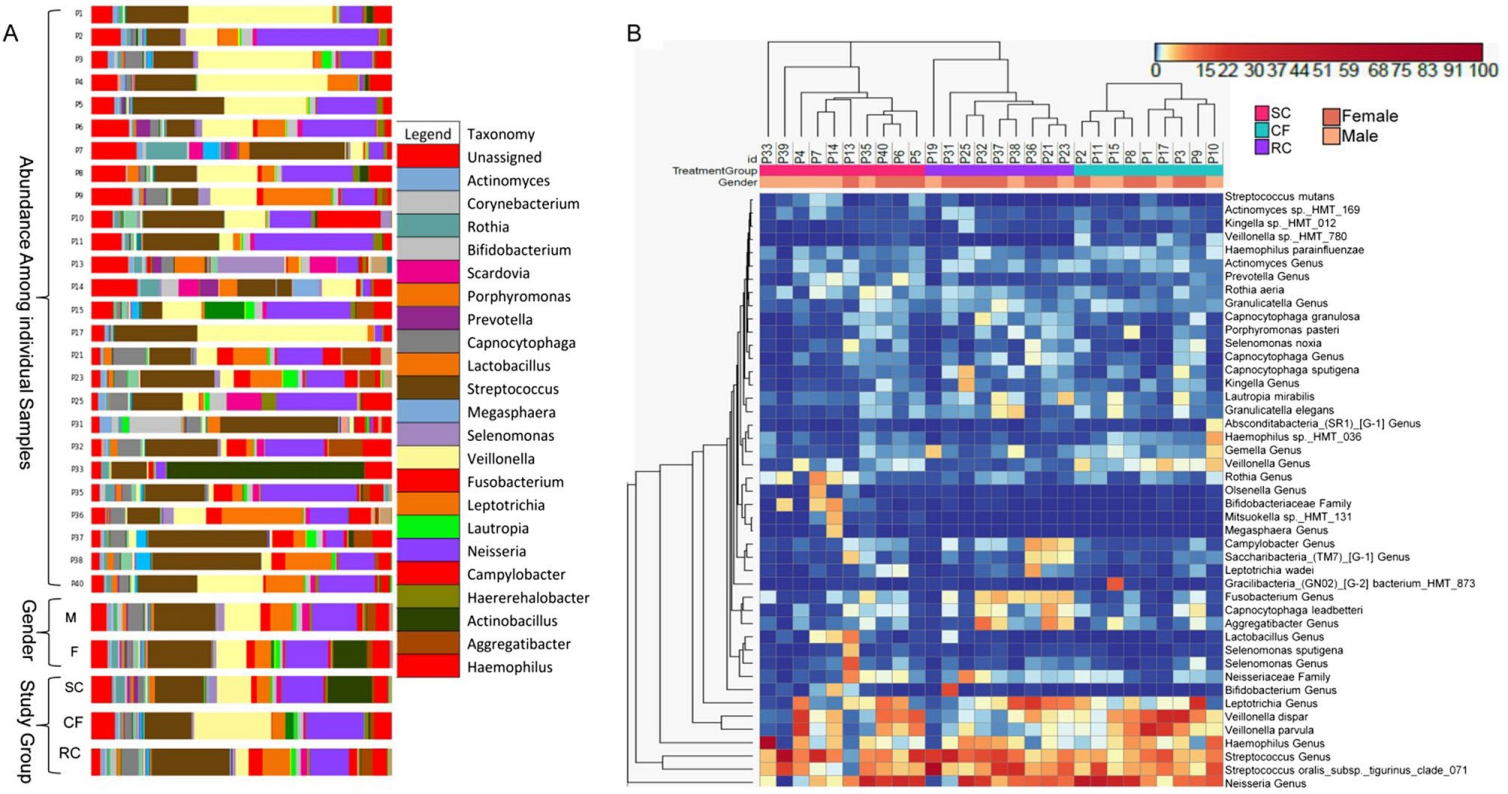
(**A**) Broad and Fine detail compositional differences of microbiota at genus level. (**B**) Coloured heatmap represent the hierarchical clustering of microbiota data at a bacterial family level. Abundances are coloured according to the colour key on the top left with blue representing a value of zero. Euclidean distance and complete linkage were used to cluster the rows and columns of the heatmap. All taxa present at less than 1% in all three groups were excluded from the heatmap. Heatmap was annotated using plotly_4.8.0.

Upon aligning the 52,628 unique OTU’s with the HOMD database at a 97% similarity level a total of 780 species from 10 phyla were classified. Percentage distribution of relative abundance of bacterial diversity at genus level was given in Fig. 2A. Among the 5 most abundant phyla *Firmicutes* dominated in all the three groups (CF—46.56%, SC—3.25%, RC—42.02%) followed by *Proteobacteria* (CF—36.99%, SC—32.98%, RC—29.55%). The next dominant group was *Actinobacteria* followed by *Fusobacteria* in SC, whereas CF was dominated by unassigned (9.3%) and RC was dominated by *Fusobacteria* (12.43%) and *Bacteroidetes* (6.43%). The abundance level of *Actinobacteria* was diminished in CF with 2.6% and it was slightly higher in RC with 5.1%. The Candidate Phyla Radiation (CPR) phyla TM7 was found to occur in all three groups with low abundance whereas SR1 and GN02 were represented only in the CF group. All 10 phyla constituted around 93.3% of aligned taxonomy while the remaining 6.63% constitute unassigned bacteria (Fig. S1). Coloured heat map depicts the phylogenetic relatedness based on Euclidean distance between samples and their constituent microbial taxa that are represented by vertical and horizontal dendrogram (Fig. 2B). Horizontal clustering clearly exhibits the grouping of taxon based on their relative abundance in every individual with major clusters of *Neisseria, Streptococcus, Haemophilus, Veillonella and Leptotrichia*.

### Over-represented and under-represented bacteria in SC and RC

To understand the over and underrepresented bacteria in the tooth biofilm of each study group, top 100 taxa based on their relative abundance was subjected to Two-way ANOVA with multiple comparisons among the study groups. High relative abundance of *Streptococcaceae, Neisseriaceae, Pasteurellaceae, Veillonellaceae, Leptotrichiaceae* and low relative abundance of *Corynebacteriaceae* was observed to be common in all three study groups. In SC samples *Micrococcaceae, Selenomonadaceae, Bifidobacteriaceae, Lactobacillales* were overrepresented and *Absconditabacteria_(SR1)_[F1], Fusobacteriaceae, Porphyromonadaceae, Campylobacteraceae* were represented with low relative abundance. The CF group is overrepresented by *Gemellaceae, Flavobacteriaceae* with a high relative abundance and *Gracilibacteria_(GN02), Absconditabacteria_(SR1), Campylobacteraceae, Prevotellaceae,* with low abundancy level. Families like *Fusobacteriaceae, Campylobacteraceae,* are expressed at high abundance in RC, whilst it exhibited low to very low abundance in SC and CF group. RC group show relatively low abundance in families like *Gemellaceae, Absconditabacteria_(SR1)_[F1], Prevotellaceae, Lactobacillaceae, Corynebacterium, Selenomonadaceae.* Significance of high abundant bacteria at genus level between the study groups was depicted in (Fig. S2). *Streptococcus oralis* subsp. *tigurinus* clade 071 and other non-mutan *Streptococcus* exhibit a highly significant difference in SC and RC group from that of the CF group. *Fusobacterium* was found to be significantly very low in the CF group than in the SC and RC groups. *Veillonella parvula* and *Veillonella dispar* were reported in the CF group with a highly significant difference from that of RC and SC group. Of *Neisseria* and *Haemophilus* that occur high in CF, *Neisseria* was found to be highly significant than that of *Haemophilus* from RC and SC group. Other caries causing bacteria like *Leptotrichia*, *Aggregatibacter, Capnocytophaga leadbetteri*, *Selenomonas*, *Prevotella*, *Rothia* found in high occurrence in SC and RC group when compared to the CF group with no significant difference among them.

### Diversified bacteria that represents potential biomarkers in caries and caries-free conditions

**Figure 3:**
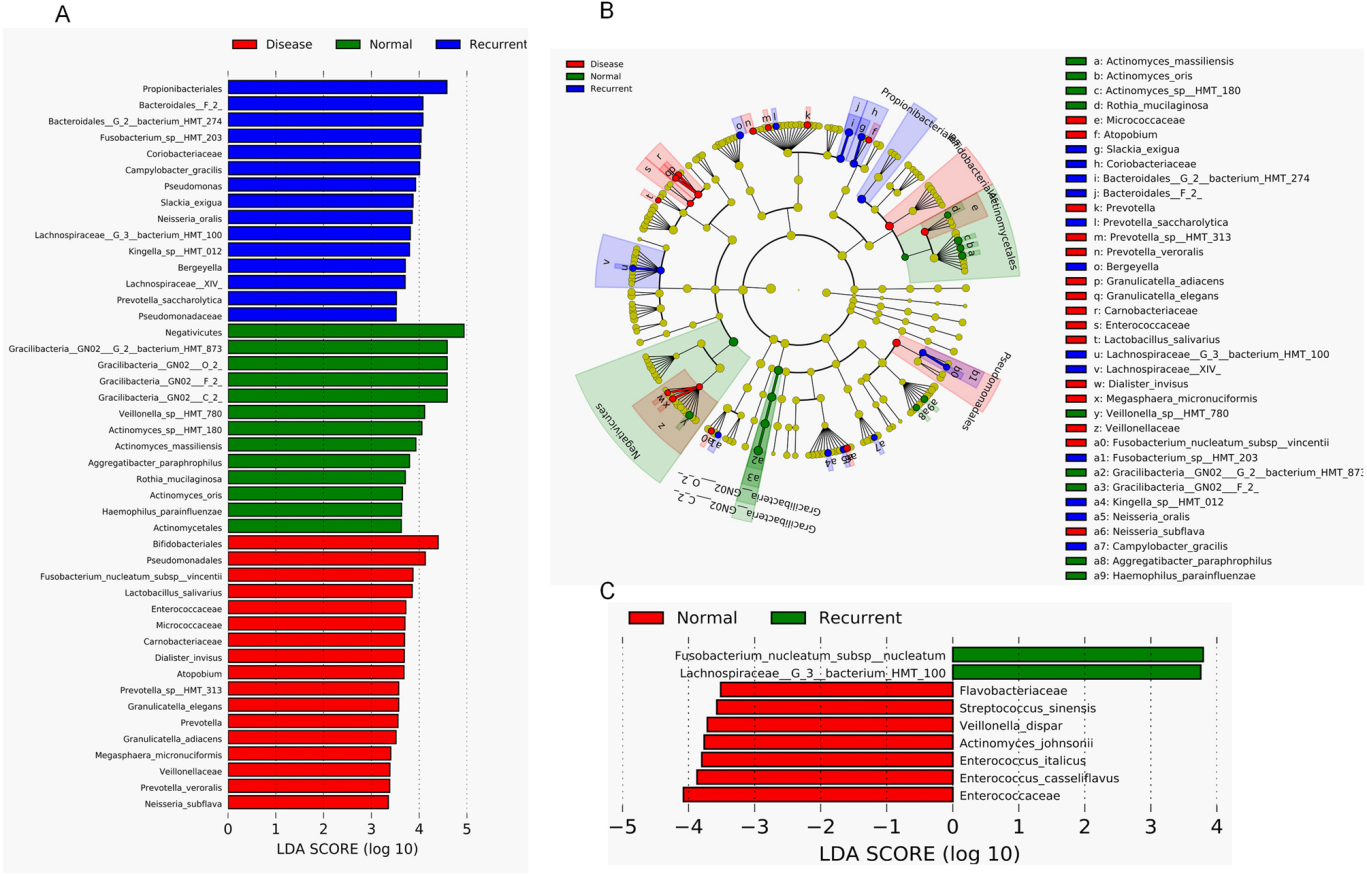
The potential biomarkers defined by LeFSe analysis and LDA (**A**–**C**). Cladogram for the taxonomic representation of significant differences between CF, SC and RC groups. The coloured nodes from the inner to the outer circles represent taxa from the phylum to genus level. Histogram of the LDA scores for differentially abundant features among groups. The threshold on the logarithmic LDA score for discriminative features was set to 3.0. (**A**) Histogram of the LDA scores (log 10) computed for features that were differentially abundant in children with SC, RC and CF. (**B**) Taxonomic representation of statistically and biologically consistent differences in children with SC, RC and CF. (**C**) Histogram of the possible biomarkers between RC and CF. https://huttenhower.sph.harvard.edu/galaxy/.

Unique bacterial community composition associated with the tooth biofilm was examined by comparing the relative abundance of the taxa among SC, RC and CF groups by LeFse analysis. The SC group is sturdily represented by 17 differentially abundant taxa that include *Bifidobacteriales, Pseudomonadales, Fusobacterium nucleatum* subsp. *vincentii, Lactobacillus salivarius, Enterococcaceae, Micrococcaceae, Carnobacteriaceae, Dialister invisus, Atopobium, Prevotella sp_HMT_313, Granulicatella adiacens, Granulicatella elegans, Megasphaera micronuciformis, Veillonellaceae, Prevotella* and *Neisseria subflava*. In recurrent groups the differences were attributable to the enrichment of *Propionibacteriales, Bacteroidales_G_2_bacterium_HMT_274, Fusobacterium *sp*._HMT_203, Coriobacteriaceae, Campylobacter* *gracilis*, *Pseudomonas, Slackia exigua, Neisseria oralis, Lachnospiraceae_G_3_bacterium_HMT_100, Kingella *sp*._HMT_100, Prevotella saccharolytica and Bergeyella.* Interestingly the tooth biofilm of the CF group was dominated by *Negativicutes* followed by four different *Gracilibacteria_GN02,* three different *Actinomyces *sp*., Veillonella *sp*._HMT_780, Rothia mucilaginosa and Haemophilus parainfluenzae* (Fig. 3A). Thus, the significant differences observed in the abundance of *Bifidobacteriales, Granulicatella, Micrococcaceae, Carnobacteriacea, Enterococcaceae* and *Lactobacillus* between the CF and SC groups may be related to the disease condition in affected children. Additionally, the significant differences observed in the abundance of *Propionibacteriales, Pseudomonadales, Lachnospiraceae, Bergeyella, Prevotella, Slackia* could be related to the secondary caries formation in RC groups (Fig. 3B). It has been observed that *Fusobacterium nucleatum and Lachnospiraceae_G_3_bacterium HMT 100* might be the key organism in the severity of the disease conditions (Fig. 3C).

### Mutual relationship among biofilm forming caries microbiota exhibited in SC and RC ecological niche

**Figure 4:**
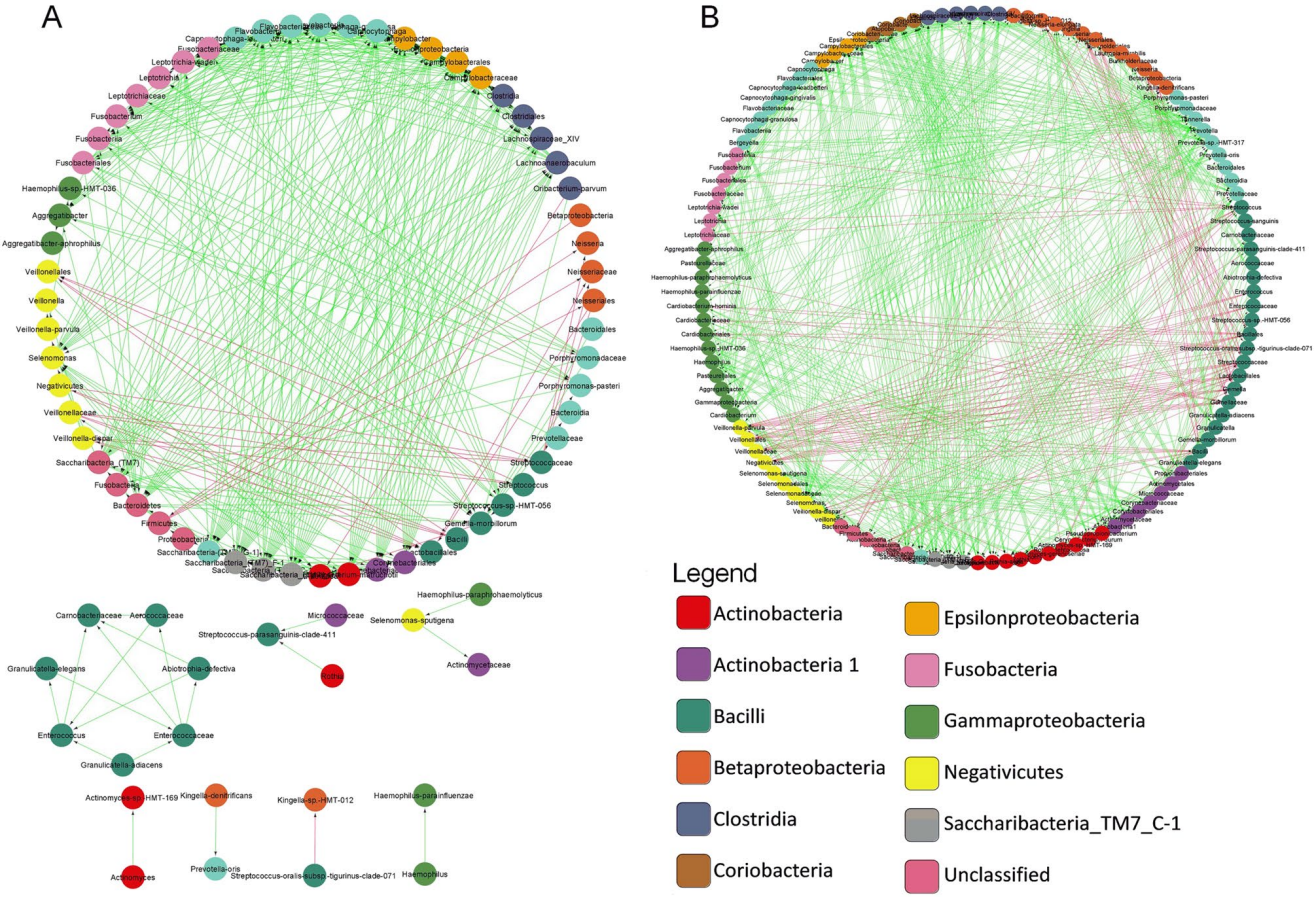
Co-occurrence interaction networks of oral microbial communities is shown in the above figure. (**A**) The complete network of core microbiome that represented in 95% of the all three study group. (**B**) The significant central interaction in recurrent caries. The solid green line represents co-presence and dotted red line indicates mutual exclusion. Arrows at the end of each line represent the direction of interaction among the different microbes. All the interacting microbes are represented with different colour according to their Class.

Co-occurrence network among the core microbiome was constructed with the organisms that occur in 90% of the study group which consists of 80 nodes and 357 edges. Both positive and negative correlation among the interacting groups were shown as edges with green and red colour respectively. This network was characterized with two major clusters one with early colonizers and other with middle and late colonizers. All the late colonizers were found to interact mutually with each other. Separate clusters among the bacteria *Carnobacteriaceae, Aerococcaceae, Granulicatella elegans, Enterococcus* and *Abiotrophia defective* that belongs to the class *Bacilli* were observed. (Fig. 4A). The network of disease conditions constitutes 127 nodes with 2115 interactions indicating a strong association of complex networks among the bacterial community. Analysis on interacting microbes reveals that the *Coriobacteria, Micrococceae* was found to occur exclusively in caries condition exhibiting positive interaction with most of the early and late colonizers. *S. oralis* subsp*. tigurinus* clade 071 that shows high abundance in all three study groups had negative interaction with *Kingella*_sp*._HMT_012* in the core microbiome. However, in the disease network *S. oralis* subsp*. tigurinus* clade 071 exhibited positive interaction with *Streptococcus, Carnobacteriaceae, Bacillales, Gemella, S. parasanguinis* and *Granulicatella* (Fig. 4B). Central interaction mainly occurs among *Corynebacterium durum*, *Haemophilus parainfluenzae*, *Oribacterium*, *Enterococcaceae, Micrococcaceae, Capnocytophaga sputigena*, *Capnocytophaga leadbetteri*, *Prevotella* and *Lachnospiraceae*. The interaction that occurs among *Pasteurellaceae*, *Corynebacterium matruchotii*, *Eikenella*, *Fusobacterium*, *Veillonella tobetsuensis* and *Bifidobacterium dentium* shows positive co-occurrence and *Veillonella parvula* and *Gemella* shows mutual exclusion with *Bifidobacterium dentium* (Fig. S3). Under disease condition three different strains of *Saccharibacteria_TM7_(G-1,C-1,F-1)* found to interact with most of the middle and late colonizers like *Selenomonas, Tannerella, Prevotella, Eikenella, Lachnoanaerbaculum, Clostridia, Camphylobacter, Epsilonproteobacteria, Capnocytophaga* and *Aggregatibacter* positively while there seems to be no interaction among the three strains of *Saccharibacteria_TM7* (Fig. S4). *Gracillibacteria GN02* was found to have mutual interaction with the majority of biofilm forming microbes in caries free condition.

### Functional prediction of the predominant taxa among three study groups

**Figure 5:**
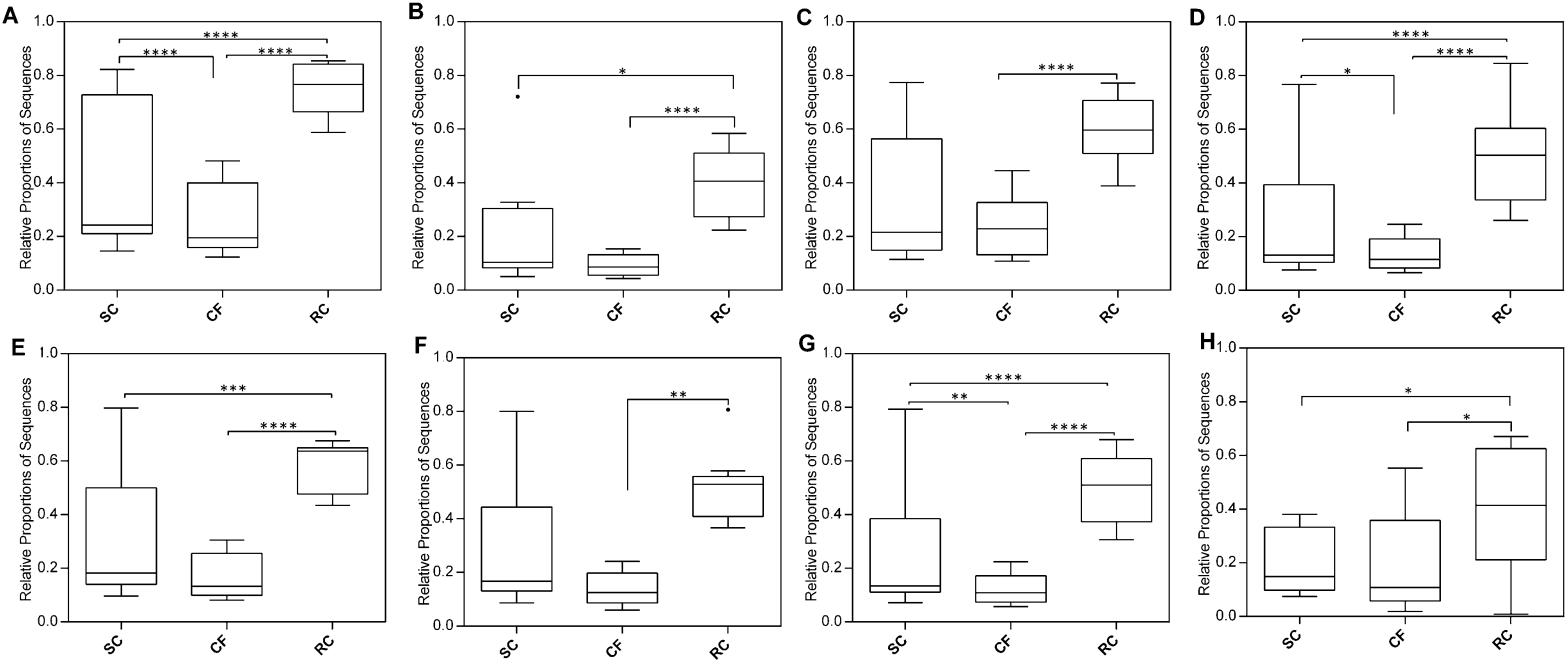
Box plot showing the significantly different KEGG pathways among the three treatment groups CF, SC, and RC. (**A**) ABC transporters, (**B**) phosphotransferase system (PTS), (**C**) bacterial secretion, (**D**) bacterial toxin, (**E**) sugar metabolism, (**F**) starch and sucrose metabolism, (**G**) galactose metabolism, (**H**) N-glycan biosynthesis. Statistical significance was evaluated using Tukey’s multiple comparisons test. P-values: * < 0.05, ** < 0.01, *** < 0.001, **** < 0.0001. Outliers are plotted as circles.

Phylogenetic Investigation of Communities by Reconstruction of Unobserved States (PICRUSt) was used to understand and infer the metabolic interactions between the microbiota of CF, SC and RC groups. Essential carbohydrate metabolisms that are related to caries like starch and sucrose metabolism, amino sugar metabolism, TCA cycle, fructose and mannose metabolism, galactose metabolism, glycolysis, pentose phosphate pathway in SC and RC exhibit highly significant difference from CF group (Fig. S5A). A distinct difference was not observed in pyruvate and inositol phosphate metabolism. Analysis on cell motility related pathway shows an increased level of bacterial toxins, bacterial chemotaxis, bacterial motility protein and flagellar assembly in RC and SC groups. The major transport systems related to quorum sensing Phosphotransferase System (PTS), ABC transport system show distinct differences between RC with that of the CF group. Glycan biosynthesis which is the prime function of oral bacterial taxon towards plaque formation found to be high in the RC group (Fig. 5).

### Network analysis of bacterial community with functional traits

**Figure 6:**
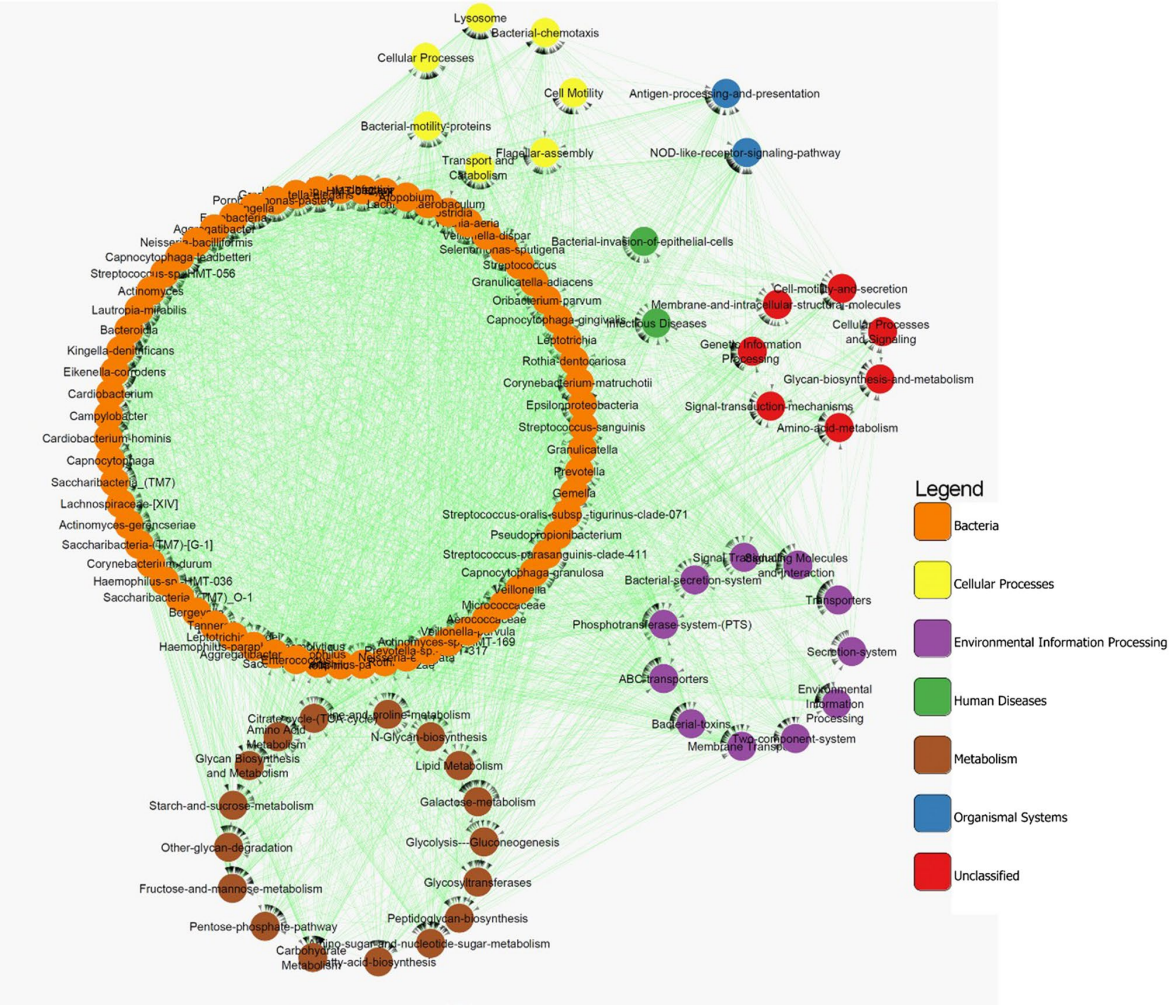
Mutual interaction networks of oral microbial communities with functional traits is shown in the above figure. The significant clustered interaction were given in different colours in recurrent caries. The solid green line represents edges with positive interactions. Arrows at the end of each line represent the direction of interaction among the interacting nodes.

Inter and intra relationship among the biofilm forming microbes and its predictive potential functional molecule was constructed to reveal the role of functional molecules for the ecological interactions among the bacteria in all three study groups. A total of 113 nodes with 2717 mutual interactions was obtained using Spearman correlation and P values were adjusted with Benjamini Hochberg multiple test correction to 0.05. More number of positive interactions was observed among different biofilm forming bacteria with that of the predicted functional traits. The network association was clustered based on the interaction among bacteria, cellular processes, environmental information processing, metabolism and organismal systems (Fig. 6). In the case of starch and sucrose metabolism we could observe a positive interaction among major bacteria like *Streptococcus, Cardiobacterium hominis, Lachnospiraceae, Selenomonas, Leptotrichia, Atopobium, Prevotella oris, Campylobacter, Coriobacteria, Rothia aeria, Actinobacteria* and *Corynebacterium*. Starch and sucrose metabolism is also positively correlated with other major functional traits like bacterial chemotaxis, pentose phosphate pathway, TCA cycle, bacterial motility proteins, flagellar assembly, N-Glycan biosynthesis, PTS, Two component system and other transport systems (Fig. S7). Of the three transport mechanism ABC transport mechanism exhibited positive interaction with almost all the carbohydrate metabolising bacteria. Along with the above mentioned bacteria in starch and sucrose metabolism *Kingella *sp.* HMT-012, Veillonella parvula, Betaproteobacteria, S. oralis *subsp. *tigurinus* clade 071*, Haemphilus* shows positive correlation with the TCS (Fig. S8). The PTS shows positive association with *Saccharibacteria-TM7, Fusobacteria, Lautropia mirabilis, Tannerella, Neisseria bacilliformis, Clostridia, Corynebacterium matruchotii, Epsilonproteobacteria, Lachnospiraceae, Gemella morbillorum, S. parasanguinis, Aggregatibacter aphophilus, Capnocytophaga* (Fig. S9).

### Comparative in vitro characterization of biofilm formation by clinical isolates and standard culture

**Figure 7:**
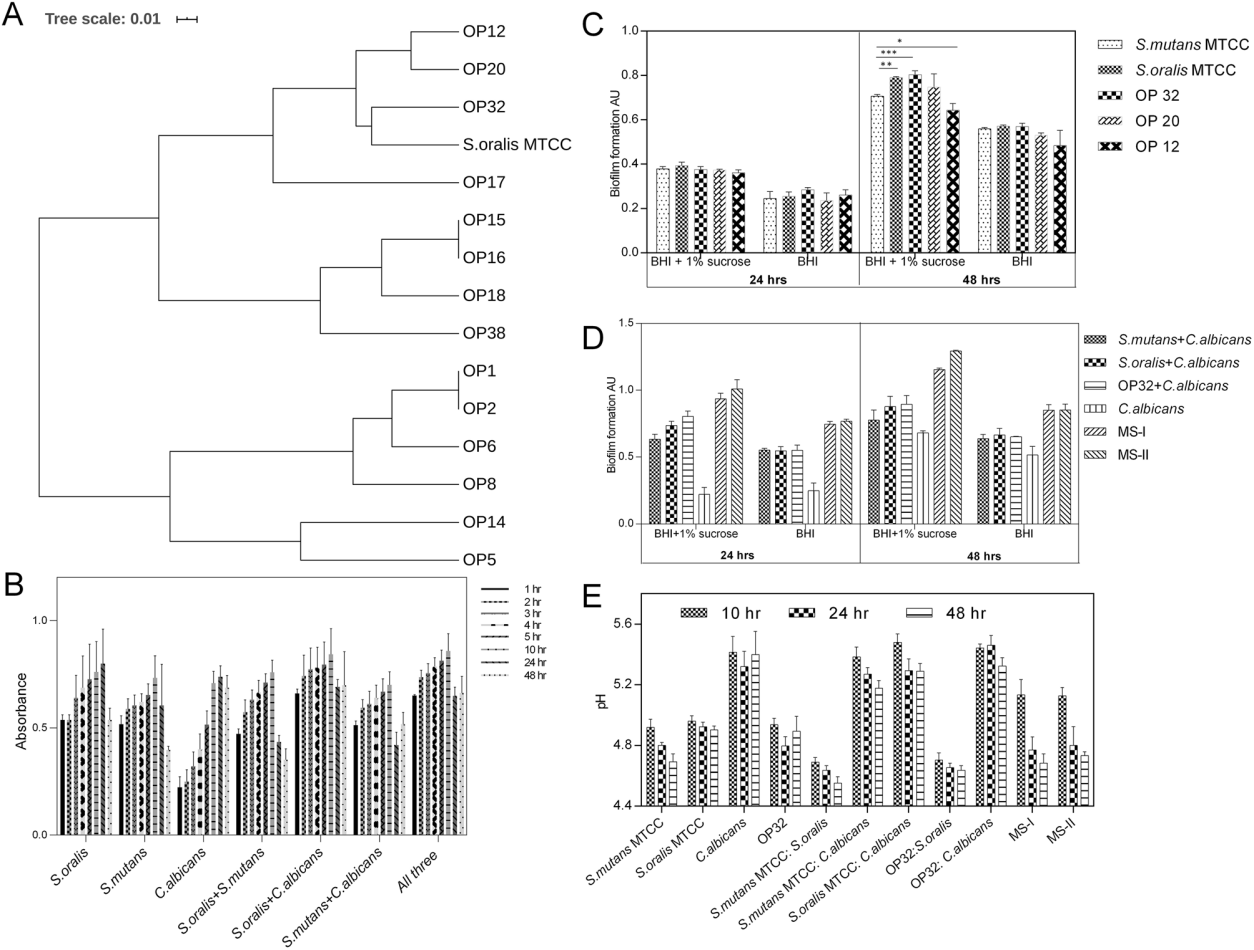
(**A**) Similarity UPGAMA tree of BOX-PCR patterns of 5 bacterial strains belonging to *S. oralis* isolated from SECC patients along with the standard *S. oralis* MTCC culture. (**B**) Biomass production assay for standard MTCC cultures both planktonic and multispecies in BHI broth for various time interval. (**C**) Analysis of Biofilm formation by crystal violet assay for standard and isolated culture in the presence and absence of 1% sucrose. (**D**) Comparative analysis of biofilm formation by crystal violet assay of standard culture and isolated culture OP32 in the presence or absence of *C. albicans*. MS-I (*S. oralis* MTCC + *S. mutans* MTCC + *C. albicans*), MS-II (OP32 + *S. mutans* MTCC + *C. albicans*) (**E**) Comparative analysis on effect of pH upon addition of sucrose in planktonic, dual and mixed species at 10 h, 24 h, and 48 h for both standard cultures and OP32. Statistical significance was evaluated using Tukey’s multiple comparisons test. P-values: * < 0.05, ** < 0.01, *** < 0.001.

Biofilm formation is a major step in caries plaque pathology. Colonies isolated from the tooth swab of SC showed varied levels of biofilm forming capabilities. Isolated clinical cultures grown in congo red agar showed various colour determinations ranging from dark red, red, light brown, black and dry crystalline black. In CRA, 15 isolated clinical cultures (OP 1, OP 2, OP 5, OP 6, OP 8, OP 10, OP 12, OP 14, OP 15, OP 16, OP 17, OP 18, OP 20, OP 32, OP 38) and all standard cultures showed positive results by producing dark black colonies in the agar plate indicating the production of exo-polysaccharide which is a main factor for biofilm formation. Representative CRA assay was given in (Fig. S10A). Phylogenetic comparison between the biofilm forming clinical isolates and *S. oralis* (MTCC) by BOX PCR showed 5 OPs closer to *S. oralis* grouped in the same clade. OP32 was the closest to *S. oralis* (MTCC) while OP12 and OP 29 showed branches near to the standard culture (Fig. 7A). OP32 was used for further analysis. Co-culture of CRA positive (OP32) with CRA negative along with standard colonies showed no antagonistic interaction. This indicated OP32 did not show negative growth impact on non-biofilm forming bacteria and vice-versa (Fig. S10B).

### Effect of sucrose on biofilm formation on dual and multispecies biofilm growth over 24 h and 48 h

Biofilm forming potential of the standard culture as planktonic, dual and mixed species at various time intervals were performed. In all the cultures, exponential growth was observed from 1 hr to 10 h and declined growth in 24 and 48 h culture which could have happened due to deficit in nutrient availability (Fig. 7B). When compared to the planktonic cells, the co-cultures (dual cultures) showed increased biofilm formation especially for *C. albicans* (MTCC) and *S. mutans* (MTCC). Both exhibited increased production of biomass in the presence of *S. oralis* (MTCC)*.* Similarly, significant increase in growth of biomass in mixed species was observed at all time intervals (Fig. 7B). Of the three clinical isolates that show phylogenetic similarity to *S. oralis* (MTCC) and biofilm forming capacity, OP32 was found to be significantly different from that of the standard cultures and other planktonic cultures at 48 h, however no significant difference was observed in 24 h culture in the presence of sucrose. Hence for dual and multispecies analysis we took OP32 as the reference clinical isolate that showed genetic similarity with *S. oralis* (MTCC) (Fig. 7C). After 24 h and 48 h of biofilm growth, both *S. oralis* (MTCC): *C. albicans* (MTCC) and OP32: *C. albicans* (MTCC) dual species were observed to have an increased biofilm formation compared to mono-species biofilms and *S. mutans* (MTCC): *C. albicans* (MTCC) in the presence of sucrose in both 24 h and 48 h. Significance difference in biofilm formation in multispecies at 48 h was observed. Especially at 48 h the biofilm formed by MSII (OP 32: *S. mutans* (MTCC): *C. albicans* (MTCC)) significantly differ from MSI (*S. oralis* (MTCC): *S. mutans* (MTCC) : *C. albicans* (MTCC)) with P < 0.001 (Fig. 7D).

The pH of the biofilm was observed to decline over 48 h of growth period ranging below 5 in all the planktonic and multispecies cultures in the presence of sucrose when compared to the control media. *S. mutans* (MTCC): *S. oralis* (MTCC) and OP32: *S. mutans* (MTCC) biofilms had the largest decrease in supernatant pH. In the case of *C. albicans* (MTCC) dual culture we could observe slightly elevated pH above 5 (Fig. 7E).

## Discussion

Severe early childhood caries (SC) and its recurrence (RC) is a critical health concern affecting both physical and psychological health of a child. The oral microbiome dysbiosis and formation of conducive cariogenic niche results in severe caries^16,17^. Poor oral hygiene and generalized treatment methods do not completely eradicate the caries forming bacteria that might lead to persistence of these bacteria causing recurrence in Indian children. High-throughput sequencing methods were used in this study to untap the hidden diversity^13^ and its varied abundance among CF, SC and RC children in India. In general, we observed varied diversity of bacteria among the study groups. We could observe high bacterial diversity in SC as compared to CF group contradicting earlier reports^4,18–20^ (Fig. 1B) while RC group was found to exhibit low diversity of bacteria enriched with late colonizers. Notably we also encountered very low diversity of bacteria in sample P19 from RC that was found to be enriched with Firmicutes (98.30%). The low diversity in RC is in corroboration with previous studies^18,20^. However, differences in beta diversity analysis revealed there could be varied proportion of taxa in RC form that of CF and SC (Fig. 1B). This might be the consequence of significantly higher metabolic activities of microbiota that contribute to host-microbiome interaction when compared with SC and CF, triggering its survival in the unique ecological niche in RC. However, significant correlation was not found between gender and any other oral demographic status with caries occurrence, similar to earlier finding^21^.

Most of the caries microbiome related studies are based on saliva samples^22^ because it is a patient friendly non-invasive approach and is easy to collect. However, this may not address the complete details of potential microbial community composition, as it varies at different intraoral sites due to the diversified ecological niche present in oral cavity^23^. In this study, the samples collected from the lesions of carious dentine gives an insight into the variation in diversity of bacteria as well as its notable community interaction in both SC and RC condition. To the best of our knowledge, the current study provides a preliminary snapshot of the tooth biofilm microbiome variation of Indian children affected by severe early childhood caries and its recurrence. About 80% of OTU based taxonomical identification constitutes to the core microbiome (Fig. 5A) of the study group which includes the representation from almost all the reported phyla except for *Fusobacteria* found abundance in RC and SC groups. This clearly indicates that presence of *Fusobacteira* plays a vital role in caries formation and progression. The decrease in abundance of dominant phyla like *Firmicutes, Proteobacteria* in such a way CF > SC > RC corroborated with the reported low diversity of bacteria occurring upon caries progression^19,20^. The observed correlation of interspecies between the low abundant heterogenic oral microbiome and the resultant niche pH might play a determining role in caries formation and its recurrence. Our study showed significant variations in low abundant species among all the study groups which may act as a potential biomarker. Late colonizers like *Prevotella saccharolytica, Campylobacter gracilis, Pseudomonas, Neisseria oralis, Coriobacteriaceae, Atopobium, Bergeylla, Fusobacterium HMT 203, Scardovia wiggsiae, Selenomonas flueggei, Stomatobaculum, Oribacterium parvum, Parvimonas* and *Peptostreptococcaceae* in SC and its maintenance in RC (Fig. S6) shows its capability of being key pathogens in the severity of the disease. This augments the keystone hypothesis, that relative abundance of prevalent taxon may not be strongly attributed to the progression of caries^15^. Co-occurrence network analysis exhibits positive association among these microbes and also with that of the early colonizers emphasizing their role in severity of caries, predicting an incomplete elimination of these microbes from the caries niche with current therapeutic methods^20,24^.

Among the oral commensals the dysbiosis of *Streptococcus* plays a pivotal role in caries formation and progression. However, the relative abundance of *S. mutans* was very low in our observation on Indian children, though it has been reported as a pioneer in caries forming bacteria^25,26^. We could find only 5% of *S. mutans* in the SC microbiome and 2% in RC (Fig. S1B,C) which clearly indicates the scarcity of *S. mutans* in advanced caries, as seen before^6,19,27^. Whereas the non-mutan *Streptococcus* and *S. oralis* subsp*. tigurinus clade 071* was found to be abundant in all the study groups subjected in this report, particularly high in RC (Fig. S2). The specific biochemical activities in *Streptococcus oralis* subsp. *tigurinus clade 071* is not clearly understood, however, its significant abundance in caries depicts a possible overlap in biochemical properties of acting as a flux and AI^24^ activity that helps in quorum sensing similar to *S. oralis*.

In vitro analysis of the clinical isolates on biofilm formation supports this. The clinical isolates OP32, OP12 and OP20 were found closely related to *S. oralis* (MTCC) by genetic fingerprinting analysis using BOX PCR that helps to identify the bacterial strains at species level^28,29^. Clinical isolates closer to *S. oralis* MTCC might be *S. oralis* subsp. *tigurinus *clade 071 as seen from the NGS data analysis and closer phylogenetic clade. These oral isolates are supposed to be the subgroup of *S. oralis* as seen earlier^30^. The positive growth interaction between the OP32 with the non-biofilm forming clinical isolates show that the interspecies interaction is significant in the SECC microbiome. Further, the increased biofilm formation in co-culture of *S. oralis* and OP32 with the major cariogen *S. mutans* under high sucrose environment show the cariogenic interrelation between the species, as observed earlier^31,32^. Also high pH reduction in the dual culture *S. mutans*: *S. oralis* and *S. mutans*: OP32 co-culture suggests its role in acidification of oral niche.

Additionally the interaction of interkingdom microbe *C. albicans* enhances the biofilm forming capability in both standard and clinical isolates. Recent report states that the glucans produced by *gtfR* mediates cross-kingdom interaction between *C. albicans* and *S. oralis* which in turn have positive effect on stronger biofilm matrix formation^31^. Similarly, we observed increased biofilm formation by *S. oralis* MTCC and OP32 in the presence of *C. albicans* both in dual and multispecies especially in the presence of sucrose and positively correlated with incubation time.

Increased level of starch and sucrose metabolism along with metabolites like ABC transporters, Phosphotransferase system, Two Component system in functional prediction analysis and its positive interaction with *S. oralis* subsp. *tigurinus *clade 071 (Fig. 6) supports our in vitro findings. These processes support an acidic environment that might allow growth of low abundant bacteria like *Micrococceae, Abiotrophia defectiva, Granulicaterlla elegans, Aerococcaceae* and *Carnobacteriaceae*. Moreover, the interaction network analysis on Two Component System which is one of the key player of biofilm formation (Fig. S8) clearly shows that *S. oralis* subsp. *tigurinus *clade 071 positively correlates with TCS and anaerobic late colonizers like *Capnocytphaga leadbetteri, Capnocytphaga granulosa* and *Aggrgatibacter*. These in turn may exhibit positive interaction with signal transduction mechanisms inviting other bacteria to interact with the biofilm more firmly and might cause the severity of the conditions.

However high abundance of other *Streptococcus* genera in SC and RC groups and its co-occurrence with other disease causing microbes and metabolic pathways exemplifies that the untapped species level diversity of *Streptococcus* still exists in the oral cavity that contributes to a greater extent in caries formation and progression. Based on the observations we envisage that *Streptococcus oralis* subsp. *igurinus *clade 071 initially colonize the tooth surface, with its carbohydrate metabolism with EPS production resulting in increased quorum sensing signals that invite other unidentified *Streptococcus* making the environment more acidic and further allows all the other middle and late colonizers to form a strong network that leads to demineralization and rupturing of teeth which may cause Severe early childhood caries.

*Leptotrichia* the third largest significant bacteria in RC group (Fig. S2) could be one of the key pathogens in RC because of its reported property to metabolize sucrose and its isomers with the help of PTS to lactic acid in the absence of *S. mutans*^33^. Significantly high PTS in RC group (Fig. 6) and its positive interaction with *Leptotrichia* in the network analysis (Fig. 7) in the current report gave a piece of clear evidence that *Leptotrichia* could create acidic niche, thereby inviting other acid tolerant bacteria such as *Capnocytophaga, Aggregatibacter*, *Fusobacterium*, *Campylobacter*, *Granulicatella*, *Gemella* and *Prophyromonas* (Fig. S9). Association of these bacteria develops a strong network of complex microbial associations that may resist any treatment methods resulting in recurrence.

Unusually, among other abundant species, we found a potent cariogen *V. parvula*^34–37^ high in CF individuals compared to RC and SC (Figs. 3, S2). The inability of *V. parvula* to enhance caries formation in these CF individuals could be linked with the high abundance of non-cariogenic arginolytic bacteria *Neisseria* and *V. dispar*^38^ in the CF oral microbiome that might neutralize the acidification of the niche through arginine metabolism^19^. The PICRUST analysis showed high arginine metabolism in the CF group, possibly from the profound *Neisseria* and *V. dispar* in CF (Fig. S5B). Occurrence of *Micrococcaceae* and *Bifidobacteriaceae* in SC and RC groups, and not in CF shows its intrinsic role in caries formation and especially recurrence condition. We found that *Micrococcaceae* interacts positively with most of the reported facultative anaerobes and with the major predicted carbohydrate metabolism like starch and sucrose, fructose and mannose and amino acid metabolism. This could play a critical role in metabolism of carbohydrate even in the anaerobic environment of SC children that could maintain the acidic niche. The abundance of *Bifidobacterium* in RC corroborated with its fluoride resistance property with the help of bifid shunt, a bypass pathway, under fluoride inhibition of enolase to reduce the pH^39^ that could lead to recurrent caries.

The co-aggregation of microbes is of prime importance among oral commensals dysbiosis that leads to complex network of biofilm formation resulting in caries manifestation^24^. Most of the core microbiome exhibited positive interaction with each other, indicting the microbes are mutually benefiting from each other’s metabolites. Co-occurrence network analysis showed that the interaction between microbes resulting in recurrence is not a single large hub, instead, several inter-related hubs of co-occurrence and mutual exclusion. Significant hubs like *Pseudomonas, Bergeyella* show mutual exclusion and *Neisseria* shows positive interaction with most of the bacteria. Prominent bacteria like *Actinomyces, Neisseria, Fusobacterium, Corynebacterium, Prevotella, Bifidobacteria, Olesenella, Selenomonas, Oribacterium* and *Gracillibacter* form a complete network of early, middle and late colonizers both in high and low abundant with different kinds of interactions depicting their role of heterogeneity in recurrence as well as severe caries (Fig. S3A). *V. tobetuensis,* one of the early colonizers possess very high autoinducer 2 activity, a strong quorum sensor^40^, co-aggregated to *Fusobacterium* in recurrent groups which could be a potent quorum sensing bacteria in the microbiome of Indian children especially in RC. This is supported by the increased sugar metabolism that resulted in increased Glycan biosynthesis in RC (Fig. 6) forming a strong matrix of biofilm with the help of bridging bacteria like *Fusobacteria* and *Corneybacerium* to link the early and late colonizers in the biofilm (Fig. S3B)^1,41^. A different bridging bacteria *Corynebacterium matruchotii* and *Corynebacterium durum* which produce acid from mannitol and galactose^42^ family were found high in RC. This has a long filamentous structure that acts as an anchoring site for other microbes^1,2^. Early colonizers like *Capnocytophaga leadbetteri*, *Haemophilus*, *Streptococcus* and late colonizers like *Oribacterium*, *Prevotella*, *Capnocytophaga sputigena* were interacting with *Corynebacterium* rather than *Fusobacterium*, while the abundance of *Fusobacterium* was more. This might add a demographic effect in the Indian population, as well as synergistic or competitive effect between *Fusobacteria* and *Corynebacteria* (Fig. S3).

As most of the earlier studies on dental caries have focused on the dysbiosis in oral microbiome^20,36^, wherein functional properties of these species in causing caries and its recurrence is contentious^6^. Positive interactions among the predicted carbohydrate metabolism and quorum sensing systems like ABC transport system, TCS, PTS with that of middle and late colonizers like *Prevotella, Cardiobacterium, Carnobacteriaceae, Selenomonas, Prophyromonas, Micrococcaceae, Rothia, Bifidobacterium, Corynebacterium, Oribacterium, Lachnoanaerobaculum, Actinomyces* and *Saccharibacteria (TM07)* could be correlated to the pathway of energy flow among these microbes that could enhances the severity of the condition by enriching more number of aciduric or acidogenic bacteria and evading the treatment method due to complexity of the bacteria present at that particular ecological niche. Significant difference in flagellar assembly between caries free and caries condition could be related to the abundance of late colonizer *Selenomonas* and also other flagellated organisms. Positive associations of three members of CPR phyla members *Gracillibacteria* GN02, *Absconditabacteria* SR1 and *Saccharibacteria* TM7 with that of three study groups CF and SC, RC respectively was observed. These findings are in line with the studies that proved the role of SR1 with H_2_S production^43^ and TM7 as obligate symbiont with *Actinomyces* species in oral disease conditions^44^. From this study we could infer that GN02 could be considered as a potential biomarker for the healthy status and TM7 for caries status of oral cavity (Fig. 4).

Thus, from the results, we observed the significant interaction among overrepresented and underrepresented bacteria and the functional metabolic interrelationship is essential in SC as well as its recurrence. In Indian children, we observed different combinations of middle and late colonizers in the polymicrobial system with the presence or absence of the so far reported early colonizers. This tends to develop a strong network of microbial association in caries condition that could develop resistance against most of the treatment methods and also nullifies the effect of resident bacteria that are trying to regain the acid environment which leads to the recurrent condition.

## Conclusion

Severe Early Childhood Caries is a chronic and prevalent disease that affects the overall growth of the children, and recurrence after treatment persists also in adulthood. This study reports the nature and heterogeneity of microbiome in the dentine micro-ecological niche among the healthy and caries affected children in Indian sub-populations. This will help to develop some simple and cost effective solutions to treat this disease. Increased depth of high throughput sequencing analysis allowed us to identify significant high and low abundant bacteria and its co-occurrence towards the aetiology of caries formation and its recurrence in Indian children. From our study, we understood that *S. oralis* subsp. *tigurinus* clade 071 is the prevalent and significant among caries affected Indian children instead of *S. mutans* and is a potent cariogen. Detailed investigation of *S. oralis* subsp. *tigurinus* clade 071 towards caries etiology and its inter-kingdom ecological interactions would deepen the knowledge about its persistence towards treatment method and how it evades the host immune system. The biomarker and co-occurrence analysis have given a better insight on the contribution of low abundant bacteria towards the severity of the disease. Our findings significantly mitigate the understanding of changes in bacterial profile in response to interaction with the predictive functional traits especially quorum sensing signals at different stages of childhood caries. This has opened up new dimensions into the oral microbiome at the community level under different micro-ecological niche to support future metabolomics and transcriptomic studies, coupled with functional assays, thereby contributing for the development of novel strategies to identify and manage the risk of caries in children.

## Materials and methods

### Study subjects and sample collection

The complete study was performed under informed consent from the parents concerned about the experimental and control group children following the World Medical Association Declaration of Helsinki guidelines. The Ethical Committee of K.S.R Institute of Dental Science and Research endorsed the design, protocol and informed written consent (132/KSRIDSR/EC/2016). Medically healthy children between the age group of 3–7 years who were reported between November 2016 and April 2018 at the Department of Pediatric Dentistry at K.S.R Institute of Dental Science and Research were recruited for this study. There was no genetic relationship among the individuals of both genders and they do not share a homogeneous living environment. Children treated with antibiotics within 3 months of the study visit were excluded. The dental health and hygiene of every subject in the study was assessed by a team of professional dentists satisfying the DMFT index standards^45^. A questionnaire on child’s food habits, brushing habit, caries prevalence in their family, especially intake frequency of fermentable carbohydrates excluding their regular interval of meals like breakfast, lunch and dinner were also collected.

A total of 55 children were divided into three groups, 1. No clinical evidence of caries experience with zero DMFT was considered as Caries-Free (CF): n = 15, 2. Children with lesion and decayed teeth with DMFT ≥ 4 were considered as Severe Early Childhood Caries (SC): n = 20. 3. Children with secondary caries and filled teeth for primary caries were considered as Recurrent Early Childhood Caries (RC): n = 20. After thorough dental examination of the children by the dentist, the teeth were gently wiped with cotton rolls followed by gentle air stream to avoid saliva contamination and biofilm was obtained using soft ended swab (Himedia, India) according to the prescribed manual of procedures for Human Microbiome (http://hmpdacc.org/resources/tools_pot.cols.php) with minor modifications and transported to lab within 4 h of sample collection^46^.

### DNA extraction from swab samples for metagenomic studies

Bacterial DNA was extracted using QIAamp DNA microbiome Kit (Qiagen, Hilden, Germany) according to the manufacturer’s instructions with minor modification in the pathogen lysis step to enhance better lysis Gram’s positive and Gram’s negative organisms. The enriched microbial DNAs were purified by ethanol precipitation. The DNA concentration was estimated spectrophotometrically using a NanoDrop spectrophotometer (Thermo Electron Corporation, USA), and molecular size was estimated by agarose gel electrophoresis. DNA samples that had passed the QC with NanoDrop concentration > 10 ng/µL was used for further processing. DNA samples were then stored at − 20 °C until further use.

### PCR amplification of the 16S rRNA gene and illumina sequencing

The V3 and V4 regions of 16S rRNA were amplified using PCR with primers Pro341F-5′ CCTACGGGNBGCASCAG; Pro805R-5′-GACTACNVGGGTATCTAATCC^47^. PCR was carried out in 50 μL reaction volume, including 32.5 μL of ddH_2_O, 10 μL of 10× Taq buffer, 1 μL of 10 mM dNTPs, 0.5 μL of 50 mM MgCl_2_, 2 μL each of 10 μM forward and reverse primers and 1 unit of Taq DNA polymerase (Thermo Scientific, USA) and 20 ng of DNA at the following PCR conditions: initial denaturation at 98 °C for 30 s followed by 30 cycles of 98 °C for 15 s, 66 °C for 25 s and 72 °C for 30 s and an final extension at 72 °C for 10 min. Thus, amplified products were subjected to next generation sequencing (2 × 250 bp) at Scigenome Research Foundation using Illumina Miseq platform according to the manufacturer’s instructions.

### Sequence analysis

The reads obtained from Illumina platform were filtered to discard sequences with an average Phred score > 30 and sequences containing incorrect barcodes and/or lacking primer sequences. Filtered reads were de-replicated, followed by subsequent removal of singletons and identified chimeras and subjected to further analysis using QIIME pipeline (version 1.9.1)^48^. The processed reads were then clustered at 97% similarity using UCLUST to identify the species-level Operational Taxonomic Units (OTUs) and the reads were mapped to the filtered OTUs to determine the exact count of each OTU in each sample^49,50^. To determine the bacterial genera the OTUs were BLAST against the HOMD database for human oral bacteria^51^. The species or phylotypes were identified by their HOMD identity. OTUs with less than 10 sequences were excluded from the analysis^37^. The sequences which could not be assigned to a genus in HOMD (at 97% identity) were considered as the probable candidates that might be unique or novel in the oral cavity of the Indian populations.

### Biomarker and network analysis

In order to identify the potential biomarker, LDA effect size (LEFSe) was performed to find out the differentially enriched taxa among the groups, the threshold for discriminative features was set to 3.0^52^ and the results were displayed in a cladogram and a bar graph^53^. Krona plot was performed to find out the percentage of enriched taxa between groups^54^. The functional prediction of microbiota was done with PICRUST. Only reads identified in closed reference picking were used for PICRUST analysis and aligned to the Kyoto Encyclopedia of Genes and Genomes (KEGG) data base^47^. Co-occurrence network analysis was performed to understand the inter and intrarelationship of oral microbiome with its predicted functional potentials obtained from PICRUST analysis among the study groups using CoNet^55^ and the model was visualized using Cytoscape^56^ tool version 3.7.2.

### Bacterial strains, growth conditions, Biofilm formation and quantification

The standard bacterial strains *Streptococcus mutans* 497T, *Streptococcus oralis* 2696, *Candida albicans* 1367T used in this study were obtained from the Microbial Type Culture Collection (MTCC), Chandigarh, India. All the microbial strains were cultured and maintained using culturing conditions given by MTCC. Clinical isolates were obtained from swab samples of caries affected children involved in this study in K.S.R. Dental Science and Research, Tiruchengode. The swab samples were cultured on blood agar (Himedia pvt Ltd, India) in order to isolate the alpha haemolytic Streptococcal strains. Thus isolated 40 strains were named as OP1-OP40 were cultured and maintained in Brain Heart Infusion (BHI) (Himedia pvt Ltd, India) media and stored as glycerol stock at − 80 °C for further use. To identify the biofilm forming isolates among the clinical cultures all the isolates were subjected to Congo Red Agar test as described earlier^57^. Co-culture of the clinical isolates and standard colonies were performed in BHI agar. The co-culture growth was observed at 37 °C growth for 24 h^58^. Biofilm was formed by pipetting out 10 µL of OD cultures for planktonic cells and equal concentration for dual and mixed cells on the wells of a flat-bottom tissue-culture treated 96-well microtiter plate containing 100μL of BHI broth. For some experiments the media were supplemented with 1% W/V sucrose. The plates were incubated statically for 24 h at 37 °C for biofilm formation. After incubation, the biofilm biomass by crystal violet assay and pH assay was performed as described previously^59^.

### BOX PCR

The 22-mer BOXAIR oligonucleotide was used to generate BOX-PCR profiles. Amplification reactions were performed in volume of 25 μL, containing 2 μM of BOX primer (5′-CTACGGCAAGGCGACGCTGACG-3′), 200 μM dNTP, PCR reaction buffer (10 mM Tris Hcl, 50 mM Kcl, 1.5 mM Mgcl 2) 1.5 units of Taq DNA polymerase and template DNA 5 μL of bacterial cell at 10^8^ CFU/mL. Amplification was performed using Lark thermocycler with the following PCR condition: initial denaturation step of 5 min at 94 °C followed by 40 cycles of 1 min at 94 °C, 1 min at 60 °C, and 1 min at 72 °C, and a final elongation step of 10 min at 72 °C followed by the analysis of BOX PCR fingerprint was done according to previous protocol^28^ and the tree was visualized using iTOL (Tree Of Life v1.0).

### Statistical analysis

OTU table that was created by QIIME analysis contains raw counts were normalized to a relative abundance OTU table. Using this relative abundance, similar types of taxa were aggregated at the phylum, class, order, family, genus and species level^48^. Biodiversity between classified groups were examined using non-parametric Mann–Whitney and/chi-squared, Fisher’s exact test. Significant differences in the alpha diversity indexes between the different groups (P < 0.05) were identified by Student’s t-test^46^. Weighted Unifrac distance of OTU samples were used to perform PCoA to analyze the differences among groups (beta diversity). Differences in the Unifrac distances for pairwise comparisons among groups were determined using Multiple t-test and visualized by the construction of a box and whiskers plot. The correlation was evaluated by the Spearman Correlation. The graphical representation of the results was performed by using GraphPad Prism version 6.01 (GraphPad Software, La Jolla California USA).

## Supplementary information

Supplementary Information.

## Data Availability

The raw reads generated in this study were deposited into the NCBI Sequence Read Archive (SRA) database under Bioproject Accession Number PRJNA454811.
