# Supplementary material for: Bacterial diversity and functional analysis of severe early childhood caries and recurrence in India
Source: Sci Rep. 2020 Dec 4;10:21248. doi: 10.1038/s41598-020-78057-z (PMC7718907; doi:10.1038/s41598-020-78057-z)
Supplement: Supplementary file 1 — Supplementary Information. [file 41598_2020_78057_MOESM1_ESM.docx]

**Bacterial Diversity and Functional analysis of Severe Early Childhood Caries and Recurrence in India**

Kalpana Balakrishnan ^1,3^, Puniethaa Prabhu^3^, Ashaq Hussain Bhat^3^, Saikiran S^3^,

Raj Pranap Arun^1^, Sarath Ashokan^4^, Sachin S Gunte^2^, Rama S Verma^*1^

^1^ Department of Biotechnology,

Indian Institute of Technology Madras, Chennai

^2^ Department of Civil Engineering,

Indian Institute of Technology Madras, Chennai

^3^Department of Biotechnology,

K. S. Rangasamy College of Technology, Namakkal Tamil Nadu, India

^4^ Department of Pediodentistry,

K. S. R Institute of Dental Science and Research, Namakkal Tamil Nadu, India

***Corresponding author:**

Rama S Verma,

Block 1, Room No.201, Department of Biotechnology,

Bhupat and Jyoti Mehta School of Biosciences,

Indian Institute of Technology Madras,

Chennai, India, 600036,

Phone: 91-44-22574109,

e-mail: vermars@iitm.ac.in

A

C

B

**Fig: S1** Distribution of the predominant bacteria at the genus level, based on relative abundance of microbiome using Krona plot. A. Abundancy distribuiton of microbiome in CF group B. Abundancy distribuiton of microbiome in SC group. C. Abundancy distribuiton of microbiome in RC group. http://krona.sourceforge.net.


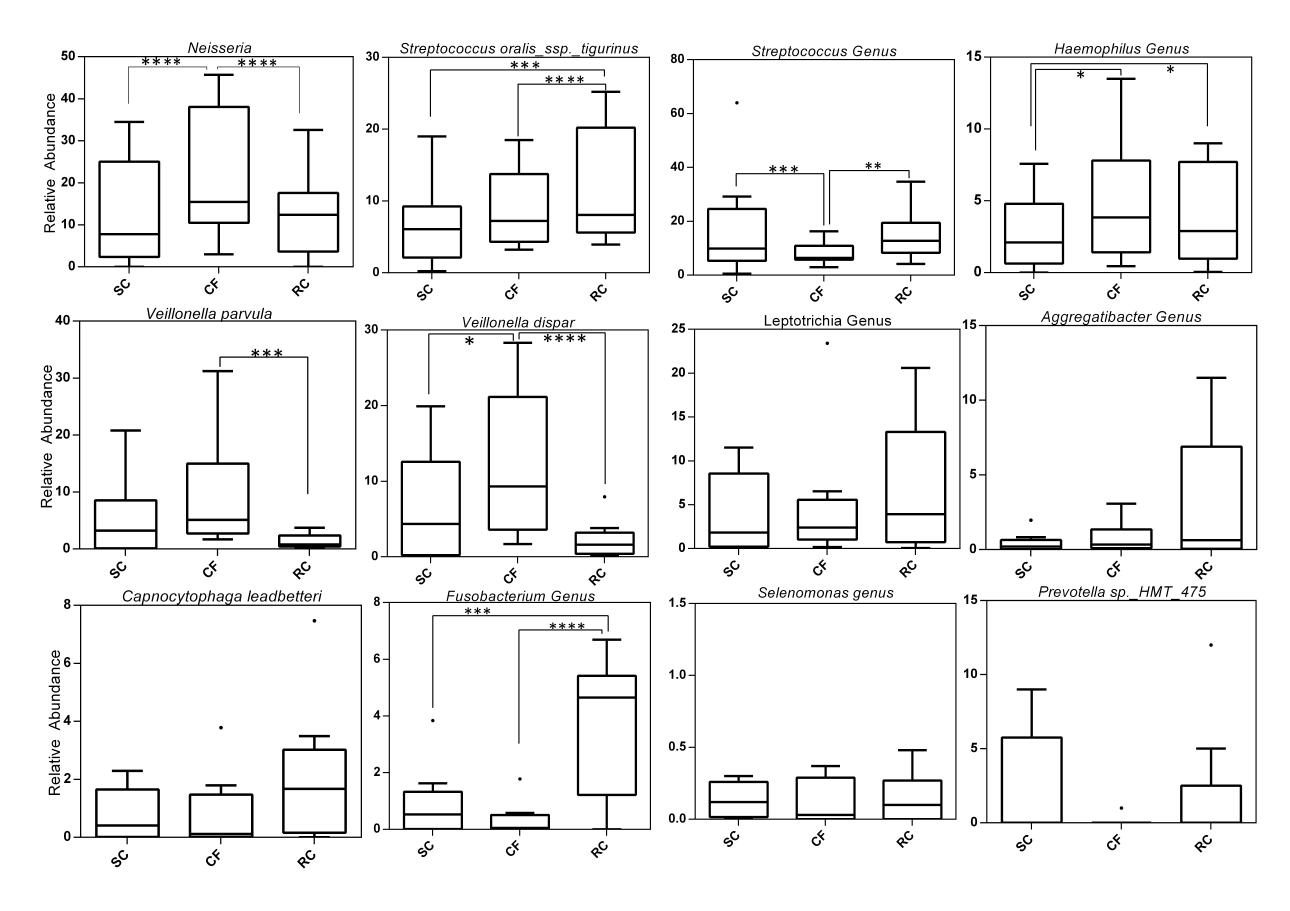


**Fig: S2** Box plots of the abundance of different taxa in the oral biofilm that shows a significant difference among the three study groups. The bottom and top of the box indicate the first and third quartiles, the line inside the box the median and the ends of the whiskers the 10th and 90th percentile values. Outliers are plotted as circles. Statistical significance was evaluated using Tukey’s multiple comparisons test. P-values: * < 0.05, ** < 0.01, *** < 0.001, **** < 0.0001

****.

**Fig: S3** Bacterial Co-occurrence interaction networks of oral microbial communities is shown in the above figure. (A) The significant central interaction in SC and RC. (B). Unique interactions of bacteria with low and high abundant microbes in SC and RC. The solid green line represents co-presence and dotted red line indicates mutual exclusion. Arrows at the end of each line represent the direction of interaction among the different microbes. All the interacting microbes are represented with different colour according to their species name


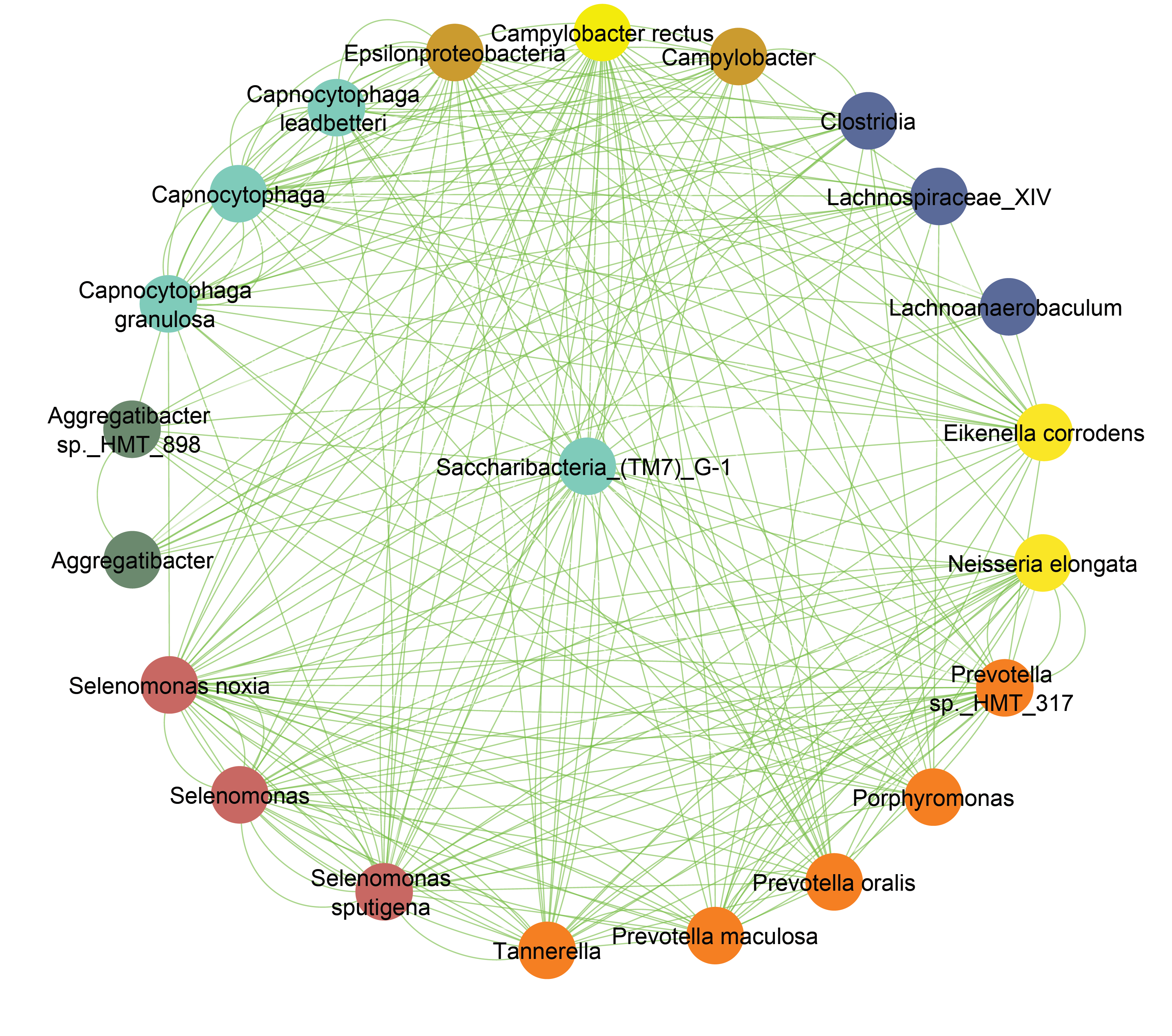

**Fig: S4** Co-occurrence networks of Saccharibacteria-TM7 with its interacting microbial communities obtained . Solid green line represents the positive interaction. The bacteria has been coloured according to their class.

**Fig: S5** A. Box plot showing the essential carbohydrate metabolism among the three treatment groups CF, SC, and RC. B. Box plot showing the Arginine metabolism among the three treatment groups CF, SC, and RC.


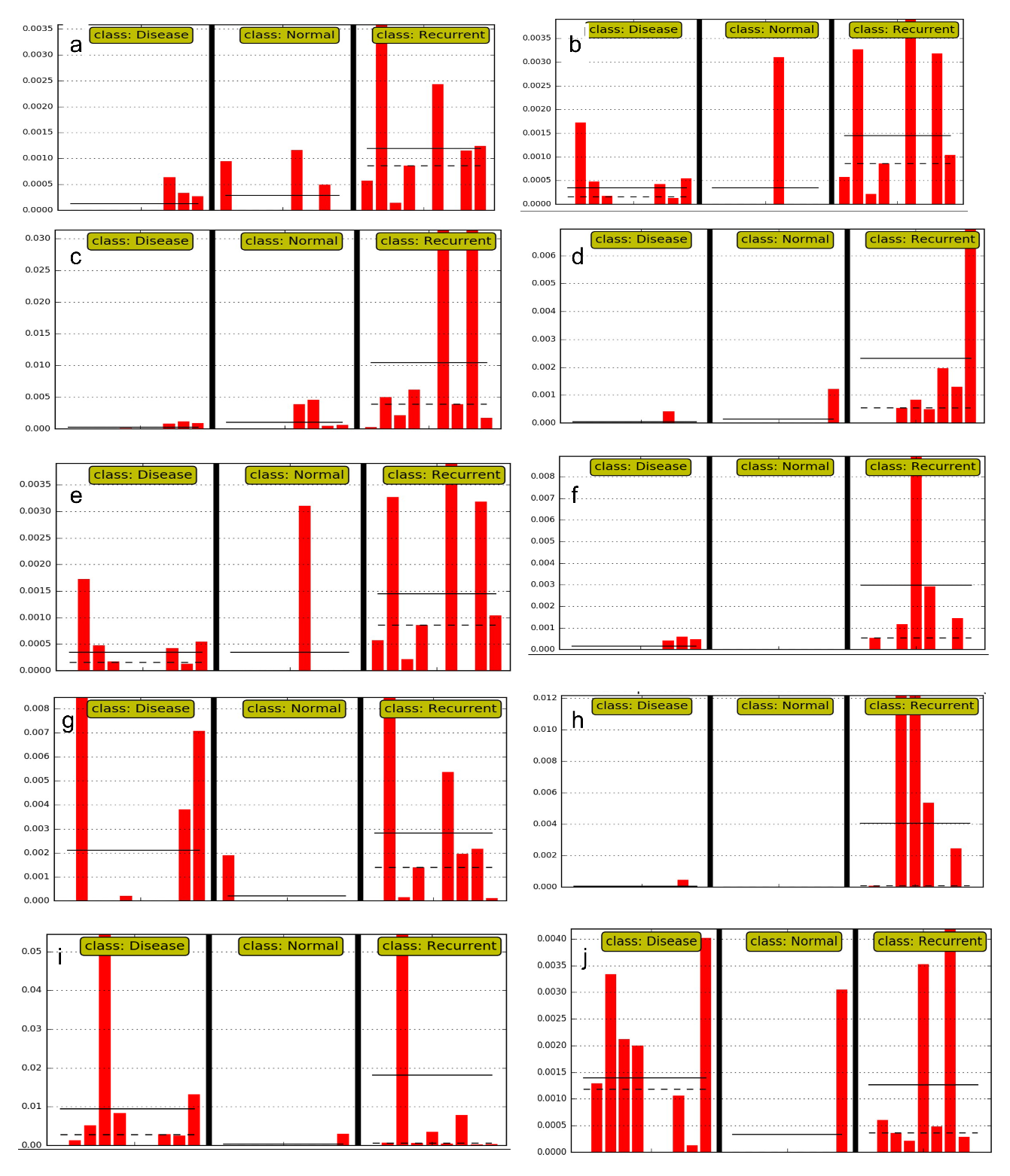


**Fig: S6** Graphical representation of relative abundance of low occurrence taxa in recurrent caries communities by LEFSe analysis https://huttenhower.sph.harvard.edu/galaxy/. Y axis is relative abundance and X axis is individual smaples. (a) *Campylobacter gracilis*, (b) *Fusobacterium HMT 203*, (c) *Bergeyella*, (d) *Pseudomonas*, (e) Lachnospiraceae__G_3_-bacterium_HMT_100, (f) *Neisseria oralis*, (g) *Prevotella saccharolytica*, (h) *Kingella p__HMT_012*, (i) *Coriobacteriaceae*, (j) *Atopobium.*


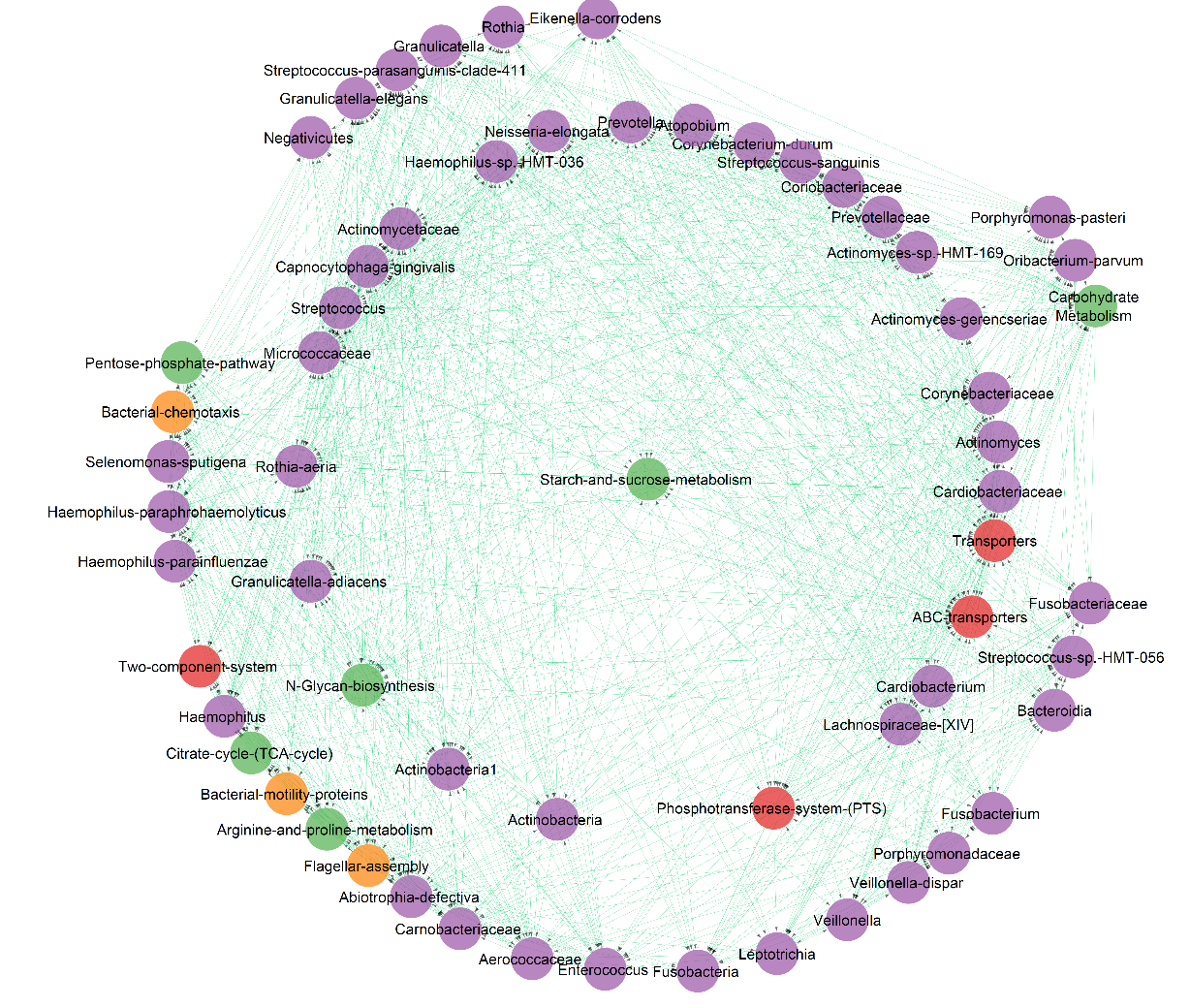


**Fig: S7** Interaction networks among bacteria and pathways related to starch and sucrose metabolism. Solid green line represent the mutual interacation and the arrow represent the target. The nodes have been coloured according to functional groups and bacteria.


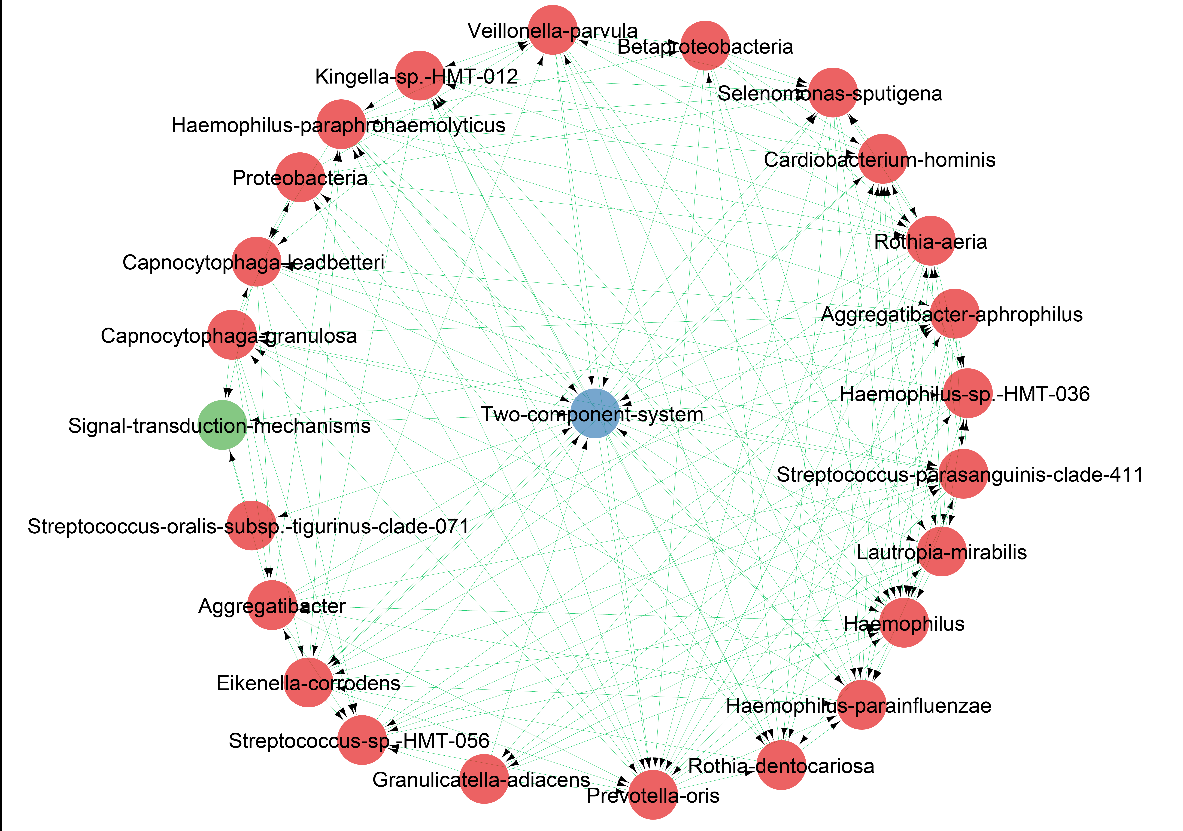


**Fig: S8** Interaction networks among bacteria and Two component system. Solid green line represent the mutual interacation and the arrow represent the target. The nodes have been coloured according to functional groups and bacteria.


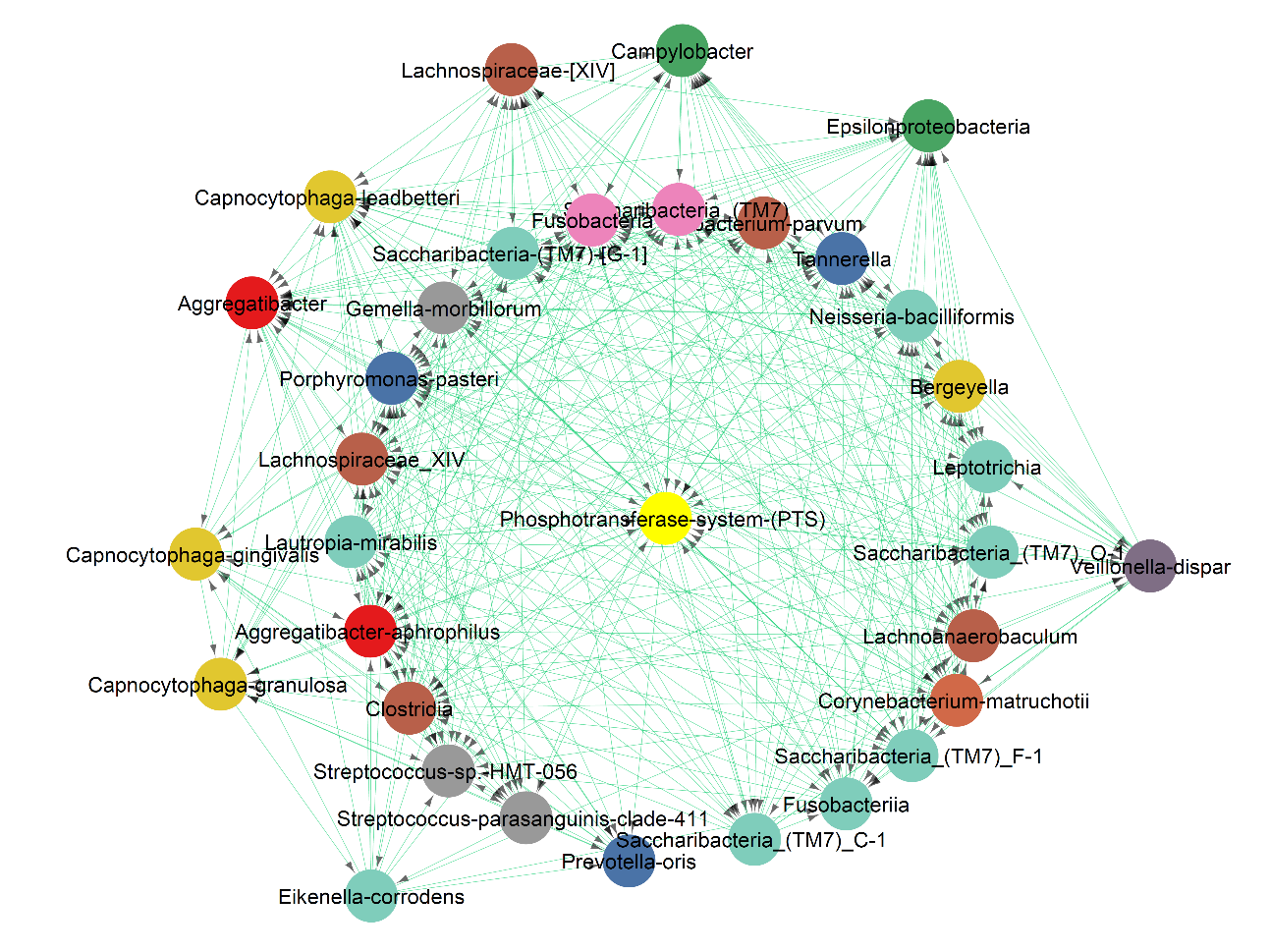


**Fig: S9** Interaction networks among bacteria and Phosphotransferase system (PTS). Solid green line represent the mutual interacation and the arrow represent the target. The nodes have been coloured according to functional groups and bacteria.

**Fig: S10 A:** Represents the Congo Red Agar test performed to isolated bacterial cultures. Dark black colonies are positive for exopolysaccharide production. **B** : Co-culturing technique depicts the synergistic effect of OP 32 (Biofilm forming culture) with non-biofilm forming culture that showed negative result in CRA test.
